# Comparing the antecedents of green computer behavior at acquisition, use, and disposal consumption stages from the moral norm and consumer attributes perspectives

**DOI:** 10.1371/journal.pone.0323622

**Published:** 2025-06-03

**Authors:** Eu-Gene Siew, Paul H.P. Yeow, Yuen Yee Yen, Wee Hong Loo

**Affiliations:** 1 School of Business, Monash University Malaysia, Selangor, Malaysia; 2 School of Business and Management, RMIT, Ho Chi Minh City, Vietnam; 3 Faculty of Business, Multimedia University, Melaka, Malaysia; 4 Department of Marketing, Business School, Sunway University, Petaling Jaya, Malaysia; Xuzhou University of Technology, CHINA

## Abstract

Computers can have an adverse effect on the environment throughout the consumption stages of their acquisition, use, and disposal. Previous studies have predominantly focused on specific stages of consumption, leading to limited research on the utilization of the values-belief-norm (VBN) model in conjunction with consumer attributes like environmental knowledge, habits, and self-identity to forecast green computer behavior across all three stages of consumption. Consequently, the aim of this research is to identify and compare the VBN factors and consumer attributes that influence green computer behavior during the three consumption stages. An understanding of the differences in the antecedents at each stage can help in the development of a holistic pro-environmental intervention to mitigate the harmful environmental effects of computer consumption. An analysis of the data collected from survey questionnaires administered to Malaysian computer consumers reveals that different factors are prominent at each stage of consumption. Self-identity is only important during computer acquisition, while habits are only important during computer use. Although environmental knowledge and VBN factors are significant at all phases, the effects differ at each stage. Environmental knowledge is the most important factor at the acquisition stage. As for the VBN factors, the ascription of responsibility is the most important factor at the acquisition stage, while the ascription of responsibility and awareness of consequences are the most important factors at the use stage. Personal norms are the most important factor at the computer disposal stage and are the most important predictor of computer disposal behavior. The outcomes of this research can help manufacturers, marketers, government policymakers, and academics engage with consumers to promote green computer behavior. The novelty of this research is in comparison of the antecedents of consumer green computer behavior at each stage of consumption.

## 1. Introduction

Computers significantly impact the environment at every stage of their consumption. An average computer uses more electricity than many other household appliances, such as the refrigerator [1]. This is exacerbated by the significant number of users who leave their computers powered all day) [1,2]. Furthermore, the improper disposal of computers leads to environmental contamination from many toxic elements, such as mercury used in display monitors or heavy metals used to produce internal computer components [3].

As a result, green computing has spawned a growing movement that seeks to mitigate the environmental impact of computer consumption 1, [4–6]. Green computing, which is called “green computer behavior” from the consumer perspective, is defined as behavior that minimizes pollution through the proper acquisition, use, and disposal of computers [7].

One of the questions researchers studying green computer behavior are trying to answer is what moral norm values, rational choices, and consumer attributes influence an individual’s decision to engage in pro-environmental behavior. This research highlights four critical areas that remain underexplored in the literature.

Firstly, extant research has shown that moral norm values (according to VBN theory) play a key role in shaping green computer behavior [8]. One of the dimensions of moral norms is biospheric values. Biospheric values shape how an individual assign worth and importance to the environment and is concerned with having principles and ideals that are “larger than oneself” [9]. Studies have shown that individuals who hold biospheric values in high regard tend to engage in pro-environmental behavior [10–12]. Despite the importance of biospheric values, there is a lack of research focusing on this variable and its effect on green computer behavior.

Secondly, pro-environmental behavior is complex and affected by factors beyond moral norms. Various consumer attributes such as environmental knowledge, habits, and self-identity have been found to impact pro-environmental behavior [13–17]. Although we use VBN theoretical factors in this research to help describe moral norms and predict green computer behavior, consumer attributes can constrain such behavior. For example, an individual may want to purchase a green computer but may be limited in how to do so by a lack of environmental knowledge. Thus, this research integrates the factors related to VBN factors with three of the consumer attributes - environmental knowledge, habits, and self-identity - to examine their effects on green computer behavior. These three variables are chosen because they represent different but complementary aspects of behavior change. Environmental knowledge represents the cognitive aspect [18], self-identity represents the motivational aspect [19] and habit represents the behavioral aspect [20].

Thirdly, although previous studies have elevated the understanding of green computer behavior, they have focused only on one or two stages of green computer consumption, such as use or disposal. According to Marcon et al. [21], there is a lack of papers linking pro-environmental consumer behavior with the different computer consumption stages. The extent to which pro-environmental consumer behavior is affected by factors related to VBN factors and consumer attributes and how much they differ at each stage of computer consumption (acquisition, use, and disposal) remains unknown. The antecedent factors that apply to one computer consumption stage may not necessarily apply to other stages. It is important to understand what these factors are at all stages of consumption so manufacturers can produce computers with less environmentally harmful components and so authorities can establish policies and incentives to promote green computer behavior more holistically [21–23].

Fourthly, few studies have examined green computer acquisition, use, or disposal in developing economies such as Malaysia. The applicability of findings from developed economies to a different context, such as Malaysia, is uncertain. Malaysia has a more collectivist culture, a relatively lower level of environmental knowledge, and a lower average income than developed economies [24,25]. As well, moral norm values, including biospheric values, are known to be affected by differences in culture [12]. Moreover, Malaysia provides an interesting research location, given that the country is ranked as the 30th largest computer importer in the world [26].

To address these four research gaps, the objective of this research is to examine consumer attributes and biospheric values from the VBN theory to predict green computing behavior at the three computer consumption stages of acquisition, use, and disposal in the setting of Malaysia, a developing economy. For consumer attributes, this paper examined environmental knowledge, frequent and infrequent habitual behaviors, and self-identity, as it is unclear from research undertaken to date how the influence of these attributes varies at different consumption stages.

This research addresses the following research questions:

1) To what extent do biospheric values and other VBN factors affect green computer behavior?
2) To what extent do the consumer attributes of habits, self-identity, and environmental knowledge influence green computer behavior?
3) To what extent does the influence of these factors differ at the computer acquisition, use, and disposal consumption stages?

## 2. Literature review and conceptual framework

### 2.1. Green computer behavior

Green computer behavior refers to responsible consumption behavior, which means consuming computers and ancillary goods and services without damaging the environment [27]. The definition of green computer behavior can be broadly divided into acquisition/purchase, use, and disposal behaviors. The existing research primarily dived into green computing behavior, solely on one aspect of the behaviour, either acquisition, use, disposal (e.g., [28] or generic and mixed green computing behavior (e.g., [29,30], within an organizational context, where the organizational framework and factors are largely be explored over individual-level factors. Nonetheless, Yeow and Loo [8] examined consumers’ consumption concerning green computers but were limited to the acquisition stage, also, the consumer attributes are neglected. Nash and Wakefield [31] research on Green IT behavior encompasses turning off the laptop when not needed and avoiding the use of paper and recycling technology devices, where the behavior is restricted to the use and disposal phase. This presents a gap in understanding the interplay role of individual-level factors comprising psychological factors (value, belief and norms) and consumer attributes (habits, self-identity, and environmental knowledge), specifically their impacts on three distinct consumption stages, from consumers’ perspectives. An examination of the consumers’ multi-faced green computer behavior and their motivators in emerging countries enables policymakers to develop tailored policies that promote sustainable practices across the entire consumption cycle. Similarly, manufacturers and marketers formulate targeted strategies that resonate with consumers in these regions, ultimately fostering wider adoption of green computer behavior that aligns with sustainable development goals (SDG). Therefore, this study extrapolates the consumption behaviors to cover the three aspects depicted below.

Phase 1 operationalizes the definition of green computer acquisition as the purchase of green computer products that have a minimal negative impact on the environment [32,33]. Green computer acquisition is important because the right purchasing decision can address the environmental problems computers create during their use and disposal phases, not merely during production [34]. For example, purchasing computers compliant with the Electronic Product Environmental Assessment Tool (EPEAT) would reduce the adverse carbon footprint of the product. EPEAT is a worldwide eco-label that certifies that electronic products meet pro-environmental standards. It is estimated that using EPEAT-certified computers resulted in “energy savings of over 38 trillion kWh,” equivalent to cost savings of about $4 trillion [35].

In Phase 2, green computer use is operationalized as “conserving significant amounts of energy and reducing carbon emissions when using a computer” [36]. This includes using computers that consume less power and shut down when not in use. It should be noted that a large number of computer users today do not turn off their computers when not using them [1,2]. and that the carbon footprint of computer use in the world is estimated to exceed that of the entire aviation industry [37].

Finally, in Phase 3, green computer disposal is operationalized as refurbishing and recycling computer waste [32]. Reuse means donating, giving away, or selling old or obsolete computers, while refurbishing means upgrading obsolete computers. Recycling means segregating computer components or selling or giving away used computers to collectors to ensure they reach the correct destination to produce new products from them. It is important to consider all three aspects of disposal because it reduces the quantity of toxic chemicals and heavy metals that reach landfills [38,39]. Reusing and refurbishing computers can help extend their lifespan, thereby reducing the energy and raw materials needed to manufacture new computers. Recycling helps recover valuable materials such as gold, silver, and platinum, reducing the energy needed to mine new supplies.

### 2.2. The moral norm model and VBN theory

This study utilizes the moral norm model, employing VBN theory, to analyze and interpret the processes of green computer acquisition, usage, and disposal. The VBN theory, initially developed by Stern et al. [40], was designed to elucidate pro-environmental behavior (PEB) influenced by moral factors. Green computing behavior, encompassing purchase to disposal, is considered analogous to PEB due to its potential to mitigate negative environmental impacts. The applicability of VBN theory has been validated across various PEB dimensions, including willingness to accept energy policies [41], intention to stay in green hotels [42], adoption of eco-innovation [43], and autonomous delivery vehicles [44]. However, it has not been specifically applied to the contexts of green computer acquisition, use, and disposal. This gap necessitates further investigation into its application in the current context, as the influence of moral factors may vary for each behavior due to the comprehensive nature and cost implications of these behaviors. The VBN posits that a causal chain of relationships will be described in the following sentences [45]. First, biospheric values prompt environmental concern [46]. Biospheric values reflect a deep commitment to the environment by the individual. Thus, making this value an important determinant of environmental concern [47]. Second, environmental concern increases one’s awareness of the consequences (AC) of their behavior. AC refers to individuals recognizing how their actions may impact others and the environment [33]. This awareness is important in shaping individuals’ beliefs about their responsibilities. Ascription of responsibility (AR) indicates an individual’s belief that they are accountable for the consequences of their actions [48]. Individuals who are more conscious of the negative consequences of their actions are more likely to feel a sense of moral obligation to act in ways that reduce environmental harm.

According to VBN theory, these feelings of responsibility (AR) help shape personal norms (PN) that guide pro-environmental actions. PN is an individual’s internalized sense of moral obligations and values to act pro-environmentally [49]. VBN theory states that personal norms will tend to predict pro-environmental behavior.

The existing literature on the application of Value-Belief-Norm (VBN) theory to predict environmentally sustainable computer behavior among consumers during acquisition, usage, and disposal remains insufficiently explored. Although Yeow and Loo [8] have contributed to this field by examining VBN theory in this context, their investigation primarily focused on a singular aspect: the purchase of green computers. Moreover, their study did not consider consumer characteristics beyond moral and rational factors that may influence decision-making processes. To address these gaps, this research seeks to expand the VBN model by incorporating consumer attributes, with the aim of providing a comprehensive explanation for the three distinct green computer behaviors. The subsequent sections will delineate the rationale for including each VBN and consumer attribute factor in this investigation.

#### 2.2.1. Biospheric values.

According to VBN theory three distinct values affect environmental concern, namely, egoistic, altruistic, and biospheric values [10,47] Biospheric values were chosen for inclusion in this research because respondents were required to answer three sets of questions regarding VBN survey items and their attributes at each consumption stage [50] and also asked about how egoistic and altruistic values may affect their environmental concern at the three stages of consumption would have made the survey a lot longer and fatiguing for respondents to complete.

Biospheric values were also chosen because the relationship between biospheric values and pro-environmental behavior is “robust across studies and populations” ([9]. Past studies have shown biospheric values to be an important determinant of environmental concern and behavior [47,51–54].. As biospheric values measure survey respondents’ values vis-à-vis the environment, they should logically affect respondents’ environmental concerns and behaviors ([47]. In addition, there is a lack of research that focuses solely on the impact of biospheric values on green computing behavior.

Despite the literature cited previously, the extent to which biospheric values affect the green computer behavior of consumers in the Malaysian context and how the effect varies between the three consumption stages is unclear. Therefore, the following hypotheses were formulated to examine these questions:

H1ai: Survey respondents’ biospheric values positively affect their environmental concern about green computer acquisition.

H1bi: Survey respondents’ biospheric values positively affect their environmental concern about green computer use.

H1ci: Survey respondents’ biospheric values positively affect their environmental concern about green computer disposal.

#### 2.2.2. Environmental concern.

Environmental concern refers to an individual’s concern about human activities and behaviors that can negatively impact the environment [48]. The VBN model theorizes that environmental concerns can affect an individual’s AC. For example, suppose an individual is deeply concerned about the environment. In that case, they may be aware that disposing of computers in a landfill will negatively affect the environment in terms of the leaching of toxic materials, such as heavy metals, into the soil. As such, the following hypotheses are proposed to investigate the effect of respondents’ environmental concerns at each stage of computer consumption:

H1aii: Survey respondents’ environmental concern positively affects the AC of their actions on the biosphere for green computer acquisition.

H1bii: Survey respondents’ environmental concern positively affects the AC of their actions on the biosphere for green computer use.

H1cii: Survey respondents’ environmental concern positively affects the AC of their actions on the biosphere for green computer disposal.

#### 2.2.3. Awareness of consequences (AC).

The concept of awareness of consequences means understanding the effects of human actions on oneself, other people, and non-human species [33]. In Stern’s VBN model, each value leads to a related environmental concern and then to a related AC. For example, a person with high biospheric values may have a high level of environmental concern for the environment, which may lead to a high awareness of consequences affecting the environment. Since this research focuses on examining biospheric values, AC is conceptualized as an individual’s awareness of the consequences of their behavior on nature and the environment.

The VBN model theorizes that AC will affect the ascription of responsibility (AR). For instance, if an individual is aware of the harmful effects of greenhouse gases, they may attribute responsibility for the effects to themself. Previous studies aggregated data and did not separate AC beliefs based on whether the values were biospheric, altruistic, or egoistic [45,48,55].As this research focuses on biospheric values, this research proposes an AC based on the biospheric context. Thus, this study forms the following hypotheses:

H1aiii: Survey respondents’ AC of their actions toward the biosphere positively affects their AR for practicing green computer acquisition.

H1biii: Survey respondents’ AC of their actions toward the biosphere positively affects their AR for practicing green computer use.

H1ciii: Survey respondents’ AC of their actions toward the biosphere positively affects their AR for practicing green computer disposal.

#### 2.2.3. Ascription of responsibility (AR).

Ascription of responsibility refers to an individual’s belief that they bear responsibility for the consequences of an action [40,48]. In this research, AR is a respondent’s perception that they feel responsible for greenhouse effects, environmental issues, and the depletion of energy resources by not practicing green computer behavior. According to the VBN theory, feelings of responsibility will lead to the generation of PN. A meta-analysis conducted by Putri Winingsih et al. [56] found validation for the effect of AR on PN. Furthermore, several empirical studies have validated the connection between AR and PN. For example, research has shown that AR significantly impacts PN’s related to reducing energy consumption [57], visiting green hotels [58], and adopting green information systems [59] However, it remains unclear whether the effect of AR on PN is significant in the context of green computer behavior. Thus, this study formulates the following hypotheses:

H1aiv: Survey respondents’ AR positively affects their PN for practicing green computer acquisition.

H1biv: Survey respondents’ AR positively affects their PN for practicing green computer use.

H1civ: Survey respondents’ AR positively affects their PN for practicing green computer disposal.

#### 2.2.4. Personal norms (PN).

The concept of personal norms is grounded on internal values and obligations to behave in a certain way [60]. PN is based on one’s perspective of “what is correct or wrong, and what is good or bad” (Thøgersen [40], as cited in Jansson & Dorrepaal [60]). Based on Jansson & Dorrepaal [60], PN is an individual’s feelings of a moral obligation and responsibility to perform green computer behavior. The VBN model states that PN will shape an individual’s internal obligations to perform pro-environmental actions. We, therefore, propose the following hypotheses:

H1av: Survey respondents’ PN positively affects their green computer acquisition.

H1bv: Survey respondents’ PN positively affects their green computer use.

H1cv: Survey respondents’ PN positively affects their green computer disposal.

### 2.3. Environmental knowledge

Environmental knowledge could be considered the cognitive aspect [18] and is the perception of having information about what green computer behavior means and of knowing how to perform pro-environmental green computer behavior [61].

Almost all the previous studies have indicated that environmental knowledge has a direct influence on pro-environmental behaviors ([16,62–65], and pro-environmental attitudes [30,66]. Nevertheless, a small number of studies did not find support for this direct relationship [67,68]. Paço & Lavrador [67] noted that environmental behavior might stem from normative motives among those with high environmental knowledge, while those with low environmental knowledge may have pro-environmental behavior without fully understanding the complexities. Wang et al. [68] argued that contextual factors may affect this relationship, and that environmental knowledge may go through other mediating factors to influence environmental behavior. Thus, the current research will test this relationship in this context in the following hypotheses:

H2a: Survey respondents’ environmental knowledge positively affects their green computer acquisition.

H2b: Survey respondents’ environmental knowledge positively affects their green computer use.

H2c: Survey respondents’ environmental knowledge positively affects their green computer disposal.

### 2.4. Habits

According to Thøgersen and Møller [20], the formation of habits is based on three behavioral requirements. First, “the behavior needs to be repeated” [20]. Behavior that is repeated annually, or once every one to two years, is infrequent habitual behavior. This contrasts with behavior performed regularly, such as driving, which is considered frequent habitual behavior. Behavior that has not been repeated cannot be considered habitual. Second, habits must arise in response to a specific cue. For instance, an individual may have the habit of reading a particular magazine in response to a visit to a hairdresser. Finally, habits are developed from experiencing rewards due to behavior.

Habits have positively affected green consumer behavior [15,17,69,70]. For example, household waste recycling and car use are frequent habitual behaviors performed automatically. Thus, a deliberate cognitive process is not required before engaging in such behavior. Although performing habitual behavior infrequently may not elicit an automatic response, infrequent habits can still affect and form a behavior [71,72]. For instance, green computer acquisition might be an infrequent habitual behavior performed once every one to two years when replacing an existing computer that has stopped functioning. Based on this, the current research conceptualizes habits as actions that are frequent and automatic or infrequent, in line with the propositions mentioned previously. Based on this, the following hypotheses are proposed:

H3a: Survey respondents’ habits positively affect their green computer acquisition.

H3b: Survey respondents’ habits positively affect their green computer use.

H3c: Survey respondents’ habits positively affect their green computer disposal.

### 2.5. Self-identity

Self-identity derived from performing pro-environmental actions can help an individual form a positive perception of the action and how others view it [73]. Self-identity, in this research, can be described as being known as one who practices green computer behavior [11]. one who projects a better self-image about themself when performing a pro-environmental behavior [74], and one who associates their social status with performing pro-environmental actions [75]. Self-identity represents the motivational aspects of pro-environmental behaviour [19].

The extant studies have shown how self-identity explains pro-environmental behaviors [13] such as recycling intentions [76], and recycling behaviors [77]. Self-identity mediates biospheric values and environmental behaviors [11]. Nevertheless, no research has looked at the role of self-identity in green computer behavior, particularly at the computer acquisition and use stages. Based on this, the following hypotheses are proposed:

H4a: Survey respondents’ self-identity positively affects their green computer acquisition.

H4b: Survey respondents’ self-identity positively affects their green computer use.

H4c: Survey respondents’ self-identity positively affects their green computer disposal.

### 2.6. Conceptual framework

**Fig 1:**
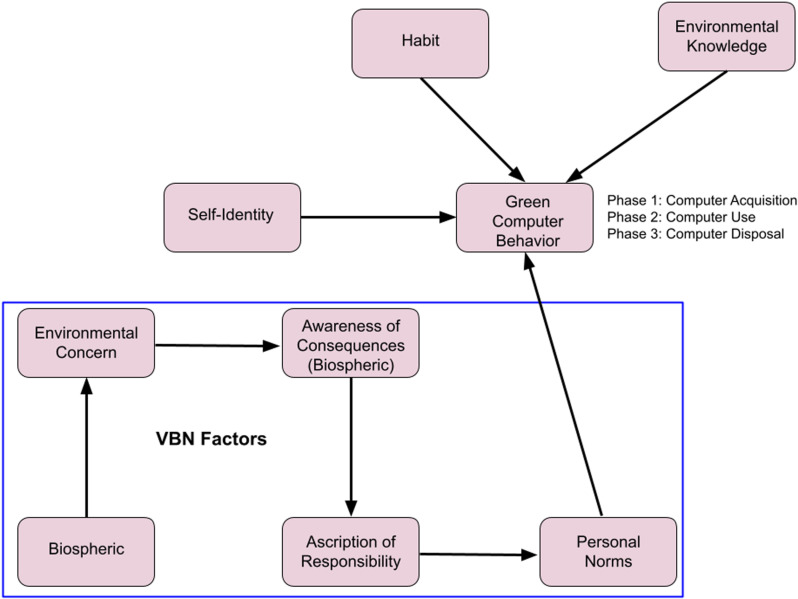
Research Framework for Phase 1, Phase 2, and Phase 3.

The hypothetical relationships among the factors in this research are shown in the conceptual framework provided in Fig 1. The figure illustrates the effects of the biospheric aspects of the VBN model (biospheric values, environmental concern, awareness of consequences, the ascription of responsibility, and the development of personal norms) and consumer attributes (self-identity, habit, and environmental knowledge) on the dependent factors of computer acquisition (Phase 1), computer use (Phase 2), and computer disposal (Phase 3).

## 3. Methodology

### 3.1. Research setting and sample

The intercept approach was used to collect research data using self-administered survey questionnaires distributed to passers-by in shopping complexes, bus stations, and parks in 13 states and 1 Federal Territory in Malaysia. Non-random convenience and purposive sampling were used because the population frame (i.e., a list of all computer owners) was non-existent. Potential respondents were informed that their participation in the survey was voluntary and that their responses and identities would be kept confidential.

As part of the selection criteria for participation in this research, respondents were asked if they had bought, used, and disposed of a computer and had a moderate understanding of green computer behavior. Respondents also had to be at least 17 years old, as users younger than this were deemed less likely to possess a computer due to their low purchasing power [78]. Each respondent who met the criteria was then asked to answer the survey questions for the three phases (Phase 1: Computer Acquisition, Phase 2: Computer Use, and Phase 3: Computer Disposal) included in the questionnaire. Respondents were asked again to confirm that they met these criteria after completing the questionnaire.

### 3.2. Measurement of the factors

S1 to S3 Appendices (A to C) show the items used to measure the factors in the three phases. The questionnaire items used in this research were adapted from previous survey instruments that had been tested and validated. The items used to measure the independent factors related to the biospheric aspect of the VBN model were adapted from Steg et al. [79]. The other independent factors, which include environmental knowledge (EKA, EKU, and EKD), were adapted from Lee [61] while the items for self-identity (SSA, SSU, and SSD) were adapted from Lee [80]. The habits (HA, HU, and HD) items were taken from Venkatesh et al. [81].

Items used to measure the dependent factor of computer acquisition were taken from Agarwal [82] and Murugesan [32]. Those used to measure computer use were from Chetty et al. [83] and Murugesan [32], while those used to measure computer disposal were from Murugesan [32] and Williams and Sasaki [84]. A Likert five-point scale, with values ranging from strongly disagree (1) to strongly agree (5), was used to measure all the items in S1 to S3 Appendices (A to C).

### 3.3. Testing of the questionnaire

Before distributing the questionnaire, ethics approval was obtained from the Monash University Human Research Ethics Committee. Before taking the survey, participants were provided with an explanatory statement. In the explanatory statement, participants were informed that they consented to participate in the research by completing the survey. They were also informed that participating in the survey was voluntary and that they could withdraw anytime.

A pilot test of the questionnaire was then undertaken with ten experts consisting of researchers and academicians, who were asked to respond to the questionnaire and provide feedback to improve clarity and provide validation of content before administration to actual respondents. Responses collected during the pilot test were not included in this final research. The survey questionnaire was then revised in response to feedback provided during the pilot test. Before the pilot test, the survey questions had been worded positively. After the test, some questions were reworded negatively to prevent respondents from giving the same responses. Additional revisions included modifying the questionnaire’s format, contents, terminology, language, and the arrangement of questions. The reliability of the questionnaire was also tested and shown to have internal consistency, as Cronbach’s alpha value was above 0.7.

### 3.4. Data collection

After the pilot test was complete, the survey was administered to respondents. A total of 1,050 questionnaires were collected from 1/2/2017–30/4/2017. Fifty questionnaires were excluded because respondents did not proceed after reading the information sheet and because it was found that respondents did not meet the selection criteria during confirmation. An additional seven questionnaires were excluded from the balance of 1,000 responses due to values that were missing from some responses. Upon final screening, 72 responses were further excluded due to respondents’ provision of straight-line responses. This left a total of 921 usable questionnaires.

Table 1 presents demographic statistics from the survey data collected. Most respondents (77.3%) were between the ages of 17 and 32. Over 80% of respondents had at least a post-secondary education, and 57.5% earned less than RM 2,000 per month. Respondents included a good mix of men (54.2%) and women (45.8%). The differences between groups for gender, age, income, and education across the three phases (Phase 1 to Phase 3) are presented in S4 Appendix (D) (Phase 1: Computer Acquisition), S5 Appendix (E) (Phase 2: Computer Use), and S5 Appendix (F) (Phase 3: Computer Disposal).

**Table 1:** Respondents’ demographic information.

Demographics	Groupings	Frequency	Percent
Gender	Male	499	54.2
	Female	422	45.8
Age (years)	17–32	712	77.3
	33–47	181	19.7
	48 and over	28	3.1
Monthly income (RM)	0–2,000	530	57.5
	2,001–4,000	221	24.0
	4,001–6,000	114	12.4
	Over 6,000	56	6.1
The highest education level attained	Secondary and below	123	13.4
	Pre-university	48	5.2
	Diploma	334	36.3
	Degree	348	37.8
	Postgraduate	37	4.0
	Others	31	3.4

### 3.5. Data analysis

The 921 usable questionnaires were then examined to determine whether they were sufficient for SEM analysis. Kline [85] suggests that the item ratio for an ideal SEM model should be at least 5:1. This study had a total of 31, 32, and 33 items for green computer acquisition, use, and disposal, respectively. Thus, the minimum number of respondents should be 165 (33 items x 5:1 ratio) survey responses. In this regard, it was concluded that the 921 usable responses were sufficient for SEM analysis [85].

The structural equation modeling (SEM) function in the Analysis of Moment Structures (AMOS) software was used to analyze the hypothesized relationships among the various constructs described in the research model [86]. This covariance-based SEM technique integrated path and factor analyses, thereby allowing multiple relationships to be examined within the research model. Each computer consumption stage was examined separately to determine whether the factors changed at each stage of examination.

## 4. Results

### 4.1. The measurement model

All the independent and dependent constructs (computer acquisition, computer use, and computer disposal) were examined separately using confirmatory factor analysis (CFA). The three final measurement models are shown in Table 2. Three items with loadings of less than 0.70, namely, RCU2, SSD4, and RCD2, were excluded from the final analysis. In examining the three involving the three computer phases, nine constructs were identified in the final measurement model. Composite reliability (CR) was well above 0.70, indicating good internal consistency for all the factors noted in the final model [86]. The AVE value was greater than 0.5, indicating adequate convergent validity.

**Table 2:** Summary of the final measurement models for computer acquisition (Phase 1), computer use (Phase 2), and computer disposal (Phase 3).

	Computer Acquisition (Phase 1)					Computer Use (Phase 2)					Computer Disposal (Phase 3)				
Construct	Items	Loadings	Sig.	AVE	CR	Items	Loadings	Sig.	AVE	CR	Items	Loadings	Sig.	AVE	CR
Green Computer Behavior	RCA1	0.778	0.001	0.612	0.825	RCU1	0.736	0.001	0.584	0.808	RCD1	0.712	0.001	0.600	0.882
	RCA2	0.832	0.001			RCU3	0.733	0.001			RCD3	0.742	0.001		
	RCA3	0.733	0.001			RCU4	0.820	0.001			RCD4	0.781	0.001		
											RCD5	0.916	0.001		
											RCD6	0.708	0.001		
Biospheric	Bio1	0.964	0.001	0.924	0.980										
	Bio2	0.960	0.001												
	Bio3	0.956	0.001												
	Bio4	0.966	0.001												
Environmental Concern	EC1	0.853	0.001	0.725	0.929										
	EC2	0.853	0.001												
	EC3	0.850	0.001												
	EC4	0.855	0.001												
	EC5	0.845	0.001												
Awareness of consequences	ACA1	0.996	0.001	0.995	0.998	ACU1	0.986	0.001	0.964	0.988	ACD1	0.991	0.001	0.974	0.991
	ACA2	0.999	0.001			ACU2	0.973	0.001			ACD2	0.984	0.001		
	ACA3	0.997	0.001			ACU3	0.986	0.001			ACD3	0.986	0.001		
Ascription of Responsibility	ARA1	1.004	0.001	0.804	0.923	ARU1	0.967	0.001	0.968	0.989	ARD1	0.991	0.001	0.879	0.956
	ARA2	0.664	0.001			ARU2	0.991	0.001			ARD2	0.817	0.001		
	ARA3	0.981	0.001			ARU3	0.994	0.001			ARD3	0.994	0.001		
Personal Norms	PNA1	0.962	0.001	0.853	0.945	PNU1	0.922	0.001	0.673	0.859	PND1	0.937	0.001	0.803	0.924
	PNA2	0.790	0.001			PNU2	0.709	0.001			PND2	0.790	0.001		
	PNA3	1.004	0.001			PNU3	0.816	0.001			PND3	0.952	0.001		
Self-identity	SSA1	0.924	0.001	0.907	0.975	SSU1	0.994	0.001	0.763	0.927	SSD1	0.930	0.001	0.823	0.933
	SSA2	0.985	0.001			SSU2	0.744	0.001			SSD2	0.833	0.001		
	SSA3	0.982	0.001			SSU3	0.992	0.001			SSD3	0.954	0.001		
	SSA4	0.916	0.001			SSU4	0.731	0.001							
Habit	HA1	0.837	0.001	0.694	0.874	HU1	0.914	0.001	0.819	0.931	HD1	0.986	0.001	0.855	0.946
	HA2	0.810	0.001			HU2	0.883	0.001			HD2	0.788	0.001		
	HA3	0.852	0.001			HU3	0.918	0.001			HD3	0.986	0.001		
Environmental Knowledge	EKA1	0.935	0.001	0.874	0.954	EKU1	0.711	0.001	0.740	0.894	EKD1	0.775	0.001	0.849	0.943
	EKA2	0.951	0.001			EKU2	0.936	0.001			EKD2	0.972	0.001		
	EKA3	0.918	0.001			EKU3	0.915	0.001			EKD3	1.001	0.001		

RCU2, SSD4, and RCD2 were excluded because their loadings were less than 0.7.

The discriminant validity of the three measurement models were tested to determine whether the constructs were distinct. The Fornell-Larcker criterion was used to assess discriminant validity. The results showed that all construct correlations were less than the corresponding square roots of the AVE values. This showed that each construct exhibited high discriminant validity. Additionally, all of the constructs fulfilled the non-multicollinearity assumption of the underlying SEM since all of the correlations were less than 0.8 [87].

### 4.2. Structural model

**Fig 2:**
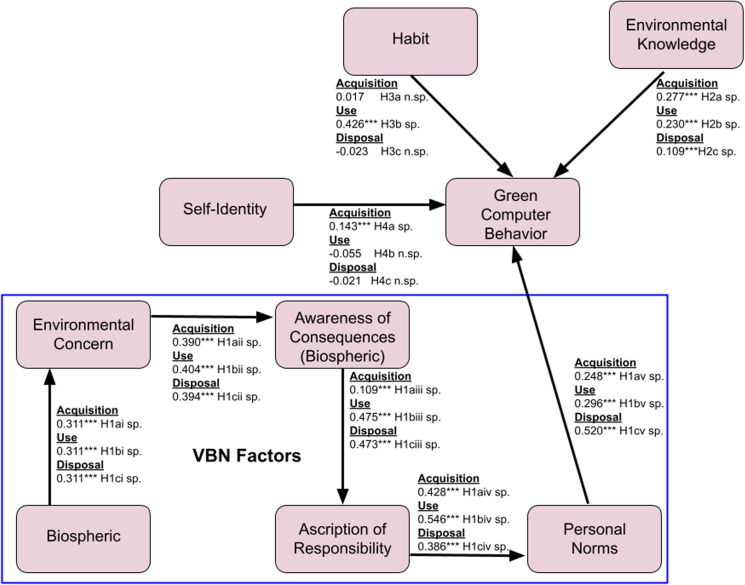
Results of the three structural models of green computer acquisition (Phase 1), use (Phase 2), and disposal (Phase 3).

This study examined the overall fit of the three structural models. The computer acquisition structural model was found to have a reasonable fit with RMSEA (0.052), with a value of less than 0.08 [88]. In addition, the GFI ([89] (0.907), and CFI [90](0.972) values were all more than 0.9, further supporting the model adequacy. The RFI value is 0.958, which is closer to 1, indicating a strong fit [88] The χ²/DF value of 4.084 was close to the acceptable maximum of 5 [91]. As for computer use, the structural model indicators showed a good fit, with the following values: RMSEA (0.055), GFI (0.909), CFI (0.969), χ 2/DF (3.787), and RFI (0.954) close to 1. Finally, computer disposal also showed a good fit with the RMSEA (0.048), GFI (0.929), RFI (0.965), CFI (0.978), and χ ^2^/DF (3.146) values. Fig 2 highlights the results of the three structural models of green computer acquisition (Phase 1), use (Phase 2), and disposal (Phase 3).

To test the hypotheses, structural model analyses were conducted separately for the three computer stages (Fig 2). The results show that environmental knowledge plays a significant role in green computer behavior for all three phases. Thus, Hypotheses H2a, H2b, and H2c are supported. Environmental knowledge has a significant impact on computer acquisition (*ß* = 0.277 and p < 0.01), computer use (*ß* = 0.230 and p < 0.01), and computer disposal (*ß* = 0.109 and p < 0.05).

However, the self-identity factor was only significant for computer acquisition (*ß* = 0.143 and p < 0.01) and not significant for computer use and disposal. As a result, Hypothesis H4a is supported but Hypotheses H4b and H4c are not. Habits were significant only for computer use (*ß* = 0.426 and p < 0.01) but not for computer acquisition and computer disposal. As a result, Hypothesis H3b is supported while H3a and H3c are not. All the VBN factors, namely biospheric values, environmental concern, awareness of consequences, the ascription of responsibility, and personal norms, were significant antecedents for green computer behavior. Thus, Hypotheses H1a, H1b, and H1c are supported.

## 5. Discussion

### 5.1. Consumer attributes (environmental knowledge, habits, and self-identity)

In comparing the three phases, some independent factors (e.g., the two consumer attributes of habits and self-identity) affected the three computer consumption stages differently. Self-identity was only significant at the acquisition stage, which aligns with Sharma et al. [92]. This reaffirmed that Malaysian consumer perceived purchasing green computers reflects their pro-environmental identity and view green computers as a premium product that may enhance their self-image. Thus, its impacts manifest in the acquisition stage, in contrast to the use and disposal stages. Habits were found to be significant only at the use stage. This study did not find support for infrequent habitual behavior at the computer acquisition and disposal stages, which is in line with Kurz et al. [70]. The habits purchasing and disposing of computers are not typically established due to the one-time or non-routine nature of the behavior at both stages. These findings contribute to the existing literature by providing evidence of the role of self-identity in computer acquisition and the role of habitual behavior in computer use.

This research sheds light on the influence of environmental knowledge on green computer behavior. Significant results were found for environmental knowledge at all three stages of green computer consumption. The results are consistent with those of many studies [16,30,66,64] but inconsistent with some others [67,68]. The results further show environmental knowledge to be the most important determinant of behavior (with the highest beta coefficient value) at the computer acquisition stage. This may be due to the context of the examined items. Green computer behavior requires the user to know about green purchases, such as if the computer being considered for purchase is EPEAT-compliant. EPEAT-compliant computers are produced with less toxic or non-toxic materials, use recycled components, are energy-efficient, and can be reassembled or upgraded easily to increase lifespan [93]. Users also need to be aware of computer settings that will reduce power consumption and that they can turn off their computers when not in use [36]. In addition, users also need to know how or where to get their computers refurbished or recycled. Nonetheless, users’ environmental knowledge may not necessarily apply to a clear-cut energy-saving situation [67]. Alternatively, users’ environmental knowledge may not directly (or indirectly, through other mediating factors) affect their intention to recycle electronic waste [68]. Thus, this research helps deepen the understanding of the role of environmental knowledge in green computer behavior.

### 5.2. VBN factors

All VBN factors are significant at all three stages. This is consistent with prior studies [55,57,94–97]. In addition, the biospheric values play an important role in predicting pro-environmental behavior. However, there are differences at each stage. At the computer acquisition stage, the ascription of responsibility is the most important factor in the framework. In contrast, at the computer use stage, the ascription of responsibility and awareness of consequences are the most important factors. At the disposal stage, personal norms are the most important factor in the VBN framework. Additionally, personal norms rather than consumer attributes are the most important predictor of computer disposal behavior.

### 5.3. Theoretical implications

This study presents two key advancements to the VBN theory. Firstly, it expands the VBN framework by adding self-identity, environmental knowledge, and habits as predictors of green computer behavior. This enhances understanding of how moral considerations and consumer attributes influence sustainable choices, addressing: (1) how biospheric values, VBN elements, habits, self-identity, and environmental awareness affect computer acquisition, use, and disposal, and (2) whether these factors vary across consumption phases. By exploring whether identical moral processes (values → beliefs → norms) apply to all green behaviors or if additional psychological factors are more relevant at specific stages, the research offers a broader theoretical grasp of sustainable decision-making. Secondly, this investigation extends the VBN theory’s application by enhancing external validity and generalizability across green computer consumption stages—purchasing, usage, and disposal. Unlike prior studies focusing on a single behavioral stage, this research provides a comprehensive view of VBN processes in diverse consumer contexts. It bolsters external validity by showing how different factors influence pro-environmental behavior at various stages; for instance, self-identity is significant during acquisition, while habits are important during use. Environmental knowledge and VBN factors are significant at all phases, but their effects vary. Environmental knowledge is crucial at acquisition. For VBN factors, the ascription of responsibility is vital at acquisition, while ascription of responsibility and awareness of consequences are significant during use. Personal norms are the primary predictor of disposal behavior. Additionally, the study enhances generalizability by extending the VBN model beyond low-cost behaviors (e.g., recycling) to include both low- and high-cost behaviors (e.g., purchasing energy-efficient computers).

### 5.4. Practical and policy implications

This research has several practical and policy implications. First, all three stages of green computer behavior are important considerations for computer manufacturers. Knowing which consumer attributes contribute to green behavior can help in the design and development of green products [14,22,23,98]. Manufacturers can use this information to promote environmental knowledge through product packaging, labeling, and instructions at the acquisition stage. They can incorporate a power-saving product feature that encourages users to develop the habit of powering down their computers by providing reminders when computers are not in use. Additionally, they can offer consumers a buy-back option during the computer purchase stage, creating environmental knowledge about how to dispose of computers in the future. This would simultaneously help consumers fulfill a moral obligation to protect the environment.

For marketing channel members, such as retailers, the focus should be on promoting environmental knowledge, self-identity, and ascription of responsibility to protect the environment at the computer acquisition stage. Marketers can educate consumers on the importance of buying green computers for a cleaner environment. The EPEAT standard should be clearly communicated since environmental knowledge is crucial for consumers who wish to purchase a green computer. Additionally, marketers should promote the social status of purchasing green computers to help consumers build a positive self-image that allows them to feel good about themselves by fulfilling their responsibility to protect the environment.

The results also benefit policymakers. In Malaysia, young consumers obtain environmental knowledge from social media [24]. Policymakers should consider developing policies, education programs, and incentives that focus on creating environmental knowledge among youth at all three computer consumption stages. This can encourage consumers to develop good habits by using the power-saving features of their equipment and recognize and boost the self-image of consumers who purchase green computers. The government can also conduct educational programs and campaigns encouraging computer refurbishment, reuse, and recycling. This can help consumers nationwide to acquire more environmental knowledge. The government can further encourage users to develop a moral obligation to practice green computer behavior through social campaigns with a focus on the following messages: (1) ascription to responsibility to protect the environment by purchasing green computers at the acquisition stage; (2) ascription to responsibility to use their computer’s power-saving mode, and awareness of the consequences (such as reducing carbon emissions) of doing so, and (3) adhering to one’s convictions/personal norms to dispose of their computer in a green manner at the disposal stage.

## 6. Conclusion, limitations, and future studies

In summary, this research identifies and compares the biospheric values, along with other VBN factors, and three of the consumer attributes affecting green computer behavior at all stages of consumption. The research framework adds consumer attributes to the existing VBN model and tests the model at each stage of consumption. Different factors are found to be significant at the various stages. Self-identity, for example, is only important at the computer acquisition stage, and consumer habits are only important at the computer use stage. Environmental knowledge and ascription of responsibility are the most important factors at the computer acquisition stage. Ascription of responsibility and awareness of consequences are the most important factors at the computer use stage, while personal norms are the most important factor at the computer disposal stage. Moreover, personal norms rather than consumer attributes are the most important predictor of computer disposal behavior.

This research has a few limitations. First, it examined only biospheric values and did not explore altruistic and egoistic values. Second, it relied on respondents to correctly and truthfully report their actual green computer behaviors since confirmation of the actual behaviour was not feasible. Third, this research is constrained by self-selection bias since a sizable number of respondents declined to participate. This paper also used the intercept approach and relied heavily on convenience sampling for data collection, which may have skewed survey responses toward a certain demographic (i.e., young respondents). Thirdly, this study focused on only three specific consumer attributes: environmental knowledge, habits, and self-identity. However, other consumer attributes, such as cultural values and psychographics, also merit further research. Lastly, this study did not specify the time frame during which the participants acquired, used, or disposed of a computer. This lack of distinction may significantly affect the results because of the shift in consumer behavior patterns over time. This is worth considering in future research.

Future research might consider breaking down the construct of self-identity into various sub-constructs, such as self-image, social image, and positive emotion. Doing so could help researchers to determine which sub-construct plays the most critical role. Another avenue of research might examine the other dimensions of VBN, which are the altruistic and egoistic values at the three consumption stages. Researchers might also consider looking at buyer acceptance of computer manufacturers incorporating a default energy-saving mode that would automatically shut a computer down when it had not been used for a specific length of time. Another interesting area of research could involve a comparison of the recycling and refurbishment of computers in Malaysia, as it would be interesting to determine the antecedent factors associated with a consumer preference to be paid by governments or NGOs to recycle or refurbish their computers to extend the useful life of their devices.

## Supporting information

S1 Appendix A. Construct measures for the computer acquisition phase. (DOCX)

S2 Appendix B. Construct measures for the computer use phase. (DOCX)

S3 Appendix C. Construct measures for the computer disposal phase. (DOCX)

S4 Appendix D. Comparing differences between groups in the computer acquisition phase. (DOCX)

S5 Appendix E. Comparing differences between groups in the computer use phase. (DOCX)

S6 Appendix F. Comparing differences between groups in the computer disposal phase. (DOCX)

S1 File Dataset. (XLSX)

S2 File Ethics release approval. (PDF)

## References

[1] Maier C. How Do Computers Pollute the Environment? 2019. Retrieved from https://sciencing.com/how-do-computers-pollute-the-environment-13660586.html

[2] Shahla A, Dahlan HM. Organizational research in the field of green IT: A systematic literature review from 2007 to 2016. Telemat Inform. 2017;34(7):1191–249.

[3] Ketchell M. Global electronic waste up 21% in five years, and recycling isn’t keeping up. 2020. Retrieved from https://theconversation.com/global-electronic-waste-up-21-in-five-years-and-recycling-isnt-keeping-up-141997

[4] Ahmaro IY, Yusoff MZBM, Abualkishik AM. The current practices of green computing approaches in Malaysia. In: Proceedings of the 6th International Conference on Information Technology and Multimedia. IEEE; 2014, 341–5.

[5] Choudhary S. A survey on green computing techniques. Int J Comput Sci Inf Technol. 2014;5(1):6248–52.

[6] Resende M, Cirillo MA. Improvement of the GFI and AGFI indexes in structural equation models by the covariance between repetitions in the observed variables. Commun Stat Simul Comput. 2024;1–15.

[7] Guemes A, Herrera P. Green computing: environmentally friendly information technology knowledge and use by university students. Int J Emerg Res Manag Technol. 2016;1:21–8.

[8] Xie J, Lu C. Relations among Pro-Environmental Behavior, Environmental Knowledge, Environmental Perception, and Post-Materialistic Values in China. Int J Environ Res Public Health. 2022;19(1):537. doi: 10.3390/ijerph19010537 35010798 PMC8744544

[9] Bouman T, Steg L, Zawadzki SJ. The value of what others value: when perceived biospheric group values influence individuals’ pro-environmental engagement. J Environ Psychol. 2020;71:101470.

[10] Nguyen T, Dekhili S. What drives responsible consumption in collectivistic developing countries? An analysis of Vietnamese consumers’ motivations with value–belief–norm theory. Bus Strat Environ. 2024;33(7):7527–43.

[11] Van Eygen E, De Meester S, Tran HP, Dewulf J. Resource savings by urban mining: The case of desktop and laptop computers in Belgium. Resour Conserv Recycl. 2016;107:53–64.

[12] Wan Q, Du W. Social Capital, Environmental Knowledge, and Pro-Environmental Behavior. Int J Environ Res Public Health. 2022;19(3):1443. doi: 10.3390/ijerph19031443 35162460 PMC8835354

[13] Gatersleben B, Murtagh N, Abrahamse W. Values, identity and pro-environmental behaviour. Contemp Soc Sci. 2014;9(4):374–92.

[14] Li L, Wang Z, Li Y, Liao A. Impacts of consumer innovativeness on the intention to purchase sustainable products. Sustain Prod Consum. 2021;27(1):774–86.

[15] Linder N, Giusti M, Samuelsson K, Barthel S. Pro-environmental habits: An underexplored research agenda in sustainability science. Ambio. 2022;51(3):546–56. doi: 10.1007/s13280-021-01619-6 34519955 PMC8800991

[16] Meng X, Tan X, Wang Y, Wen Z, Tao Y, Qian Y. Investigation on decision-making mechanism of residents’ household solid waste classification and recycling behaviors. Resour Conserv Recycl. 2019;140(1):224–34. doi: 10.1016/j.resconrec.2018.09.021

[17] Verplanken B, Orbell S. Attitudes, Habits, and Behavior Change. Annu Rev Psychol. 2022;73:327–52. doi: 10.1146/annurev-psych-020821-011744 34587780

[18] Liu S, Guo L. Based on environmental education to study the correlation between environmental knowledge and environmental value. Eurasia J Math Sci Tech Educ. 2018;14(7):3311–9.

[19] Sinickas A. Finding a cure for survey fatigue. Strategic Commun Manag. 2007;11(2):11.

[20] Thøgersen J. Norms for environmentally responsible behaviour: an extended taxonomy. J Environ Psychol. 2006;26:247–61.

[21] Marcon A, Ribeiro JLD, Dangelico RM, de Medeiros JF, Marcon E. Exploring green product attributes and their effect on consumer behaviour: A systematic review. Sustainable Prod Consum. 2022;32(1):76–91.

[22] Kang J, Moreno F. Driving values to actions: predictive modeling for environmentally sustainable product purchases. Sustain Prod Consum. 2020;23(1):224–35.

[23] Silitshena P. Self-identity is a function of a good motivational model. Int J High Educ. 2022;11(4):39–48.

[24] Ahamad N, Mariani A. Assessment of knowledge, attitude and practice towards sustainable consumption among university students in Selangor, Malaysia. Sustainable Prod Consum. 2018;16(1):88–98.

[25] Ahmad T, Bello A, Nordin M. Exploring Malaysian university students’ awareness of green computing. GSTF J Educ. 2014;1(2).

[26] Nguyen T, Lobo A, Greenland S. Pro-environmental purchase behaviour: The role of consumers’ biospheric values. J Retailing Consumer Serv. 2016;33(1):98–108.

[27] Ahmad T, Nordin M. University students’ subjective knowledge of green computing and pro-environmental behavior. Int Educ Stud. 2014;7(2):64–74.

[28] Li D, Zhao L, Ma S, Shao S, Zhang L. What influences an individual’s pro-environmental behavior? A literature review. Resour Conserv Recycl. 2019;146:28–34.

[29] Molla A, Abareshi A, Cooper V. Green IT beliefs and pro-environmental IT practices among IT professionals. Inf Technol People. 2014;27(2):129–54.

[30] Observatory of Economic Complexity. Computers in Malaysia. 2019. Retrieved from https://oec.world/en/profile/bilateral-product/computers/reporter/mys?redirect=true#market-growth

[31] Nash J, Wakefield R. The role of identity in green IT attitude and intention. J Comput Inf Syst. 2022;62(5):998–1008.

[32] Murugesan S. Harnessing green IT: principles and practices. IT Prof. 2008;10(1):24–33.

[33] Steg L, Vlek C. Encouraging pro-environmental behaviour: an integrative review and research agenda. J Environ Psychol. 2009;29(3):309–17.

[34] Leung N, Lau S, Lau S. A study of factors influencing green IT practices, buying and subscription behaviours of computer and mobile devices, and streaming services. Pac Asia J Assoc Inf Syst. 2019;11(1):4.

[35] Government of the District of Columbia, Office of Contracting and Procurement. Environmental specification guidance for computers, monitors, tablets, & mobile phones. Department of Energy and Environment; 2017. Retrieved March 10, 2025, from https://doee.dc.gov/sites/default/files/dc/sites/doee/service_content/attachments/Final%20-%20Computers%2C%20Monitors%2C%20Tablets%2C%20%26%20Mobile%20Phones%20Additional%20Information.pdf

[36] Pivetti M, Melotti G, Vespa M, Cappabianca F, Troilo F, Placentino MP. Predicting recycling in southern Italy: An exploratory study. Resour Conserv Recycl. 2020;156(1):104727. doi: 10.1016/j.resconrec.2020.104727

[37] Sekaran U, Bougie R. Research methods for business: A skill building approach. 3rd ed. ed. Wiley; 2016.

[38] Sharma N, Saha R, Sreedharan VR, Paul J. Relating the role of green self‐concepts and identity on green purchasing behaviour: An empirical analysis. Bus Strat Environ. 2020;29(8):3203–19.

[39] Uren H, Roberts L, Dzidic P, Leviston Z. High-status pro-environmental behaviors: costly, effortful, and visible. Environ Behav. 2021;53(5):455–84.

[40] Stern P. Toward a coherent theory of environmentally significant behavior. J Soc Issues. 2000;56(3):407–24.

[41] Hiratsuka J, Perlaviciute G, Steg L. Testing VBN theory in Japan: relationships between values, beliefs, norms, and acceptability and expected effects of a car pricing policy. Transp Res Part F Traffic Psychol Behav. 2018;53:74–83. doi: 10.1016/j.trf.2017.12.015

[42] Choi H, Jang J, Kandampully J. Application of the extended VBN theory to understand consumers’ decisions about green hotels. Int J Hosp Manag. 2015;51:87–95. doi: 10.1016/j.ijhm.2015.08.004

[43] Jansson J, Marell A, Nordlund A. Exploring consumer adoption of a high involvement eco‐innovation using value‐belief‐norm theory. J of Consumer Behaviour. 2011;10(1):51–60. doi: 10.1002/cb.346

[44] Chunhua J, Shuo W, Zhirong H, Li-Wei L, Jing Y. Application of the extended value-belief-norm (VBN) theory to understand consumers’ intention to use autonomous delivery vehicles (ADVs). Heliyon. 2023;9(9):e20244. doi: 10.1016/j.heliyon.2023.e20244 37809410 PMC10560012

[45] Chen M. An examination of the value‐belief‐norm theory model in predicting pro‐environmental behaviour in Taiwan. Asian J Soc Psychol. 2015;18(2):145–51.

[46] Yeow P, Loo W. Antecedents of green computer purchase behavior among Malaysian consumers from the perspective of rational choice and moral norm factors. Sustain Prod Consum. 2022;32(1):550–61.

[47] De Groot JIM, Steg L. Value orientations and environmental beliefs in five countries: validity of an instrument to measure egoistic, altruistic and biospheric value orientations. J Cross Cult Psychol. 2007;38(3):318–32.

[48] Shalabh A. Impact of green computing in it industry to make eco-friendly environment. J Glob Res Comput Sci. 2014;5(4):05–10.

[49] Stern P, Dietz T, Abel T, Guagnano G, Kalof L. A value-belief-norm theory of support for social movements: The case of environmentalism. Hum Ecol Rev. 1999;Dec(1):81–97.

[50] Sharpe R, Goodall P, Neal A, Conway P, West A. Cyber-physical systems in the re-use, refurbishment and recycling of used electrical and electronic equipment. J Clean Prod. 2018;170:351–61.

[51] Katz-Gerro T, Greenspan I, Handy F, Lee HY. The relationship between value types and environmental behaviour in four countries: universalism, benevolence, conformity and biospheric values revisited. Environ Values. 2017;26(2):223–49.

[52] Lee K. The role of media exposure, social exposure and biospheric value orientation in the environmental attitude-intention-behavior model in adolescents. J Environ Psychol. 2011;31(4):301–8.

[53] Sivapalan A, von der Heidt T, Scherrer P, Sorwar G. A consumer values-based approach to enhancing green consumption. Sustain Prod Consum. 2021;28(1):699–715.

[54] Steg L, Dreijerink L, Abrahamse W. Factors influencing the acceptability of energy policies: A test of VBN theory. J Environ Psychol. 2005;25(4):415–25. doi: 10.1016/j.jenvp.2005.08.003

[55] Ghazali E, Nguyen B, Mutum D, Yap S. Pro-environmental behaviours and value-belief-norm theory: assessing unobserved heterogeneity of two ethnic groups. Sustainability. 2019;11(12):3237.

[56] Pollard C. Applying the theory of planned behavior to individual computer energy saving behavioral intention and use at work. In: 2015 Americas Conference on Information Systems, AMCIS 2015. 2015, 1–18.

[57] Saha B. Green Computing Current Research Trends. ijcse. 2018;6(3):467–9. doi: 10.26438/ijcse/v6i3.467469

[58] Dong Z, He C, Hu T, Jiang T. Integrating values, ascribed responsibility and environmental concern to predict customers’ intention to visit green hotels: the mediating role of personal norm. Front Psychol. 2024;14:1340491. doi: 10.3389/fpsyg.2023.1340491 38259572 PMC10800668

[59] Dalvi-Esfahani M, Ramayah T, Rahman AA. Moderating role of personal values on managers’ intention to adopt green is: Examining norm activation theory. Ind Manag Data Syst. 2017;117(3):582–604.

[60] Jansson J, Dorrepaal E. Personal norms for dealing with climate change: results from a survey using moral foundations theory. Sustain Dev. 2015;23(6):381–95.

[61] Lee K. The green purchase behavior of Hong Kong young consumers: The role of peer influence, local environmental involvement, and concrete environmental knowledge. J Int Consum Mark. 2010;23(1):21–44.

[62] Amoah A, Addoah T. Does environmental knowledge drive pro-environmental behaviour in developing countries? Evidence from households in Ghana. Environ Dev Sustain. 2021;23(2):2719–38.

[63] Verplanken B, Whitmarsh L. Habit and climate change. Curr Opin Behav Sci. 2021;42(1):42–6.

[64] Wang Z, Guo D, Wang X, Zhang B, Wang B. How does information publicity influence residents’ behaviour intentions around e-waste recycling?. Resour Conserv Recycl. 2018;133(1):1–9. doi: 10.1016/j.resconrec.2018.01.014

[65] Williams E, Sasaki Y. Strategizing the end-of-life handling of personal computers: resell, upgrade, recycle. In: Kuehr R, editor. Computers and the environment: understanding and managing their impacts. Dordrecht: Springer; 2003, 183–96.

[66] Paço A, Lavrador T. Environmental knowledge and attitudes and behaviours towards energy consumption. J Environ Manage. 2017;197:384–92. doi: 10.1016/j.jenvman.2017.03.100 28410516

[67] Ojo A, Raman M, Downe A. Toward green computing practices: A Malaysian study of green belief and attitude among information technology professionals. J Clean Prod. 2019;224(1):246–55. doi: 10.1016/j.jclepro.2019.03.237

[68] Wang X, Van der Werff E, Bouman T, Harder MK, Steg L. I am vs. we are how biospheric values and environmental identity of individuals and groups can influence pro-environmental behaviour. Front Psychol. 2021;12:618956. 33679533 10.3389/fpsyg.2021.618956 PMC7930912

[69] Gkargkavouzi A, Halkos G, Matsiori S. Environmental behavior in a private-sphere context: integrating theories of planned behavior and value belief norm, self-identity and habit. Resour Conserv Recycl. 2019;148(1):145–56. doi: 10.1016/j.resconrec.2019.01.039

[70] Kurz T, Gardner B, Verplanken B, Abraham C. Habitual behaviors or patterns of practice? explaining and changing repetitive climate‐relevant actions. Wiley Interdiscip Rev Clim Change. 2015;6(1):113–28.

[71] Gardner B. Habit as automaticity, not frequency. Eur Health Psychol. 2012;14(2):32–6.

[72] Venkatesh V, Thong JY, Xu X. Consumer acceptance and use of information technology: extending the unified theory of acceptance and use of technology. MIS Q. 2012;36(1):157–78.

[73] Trudel R, Argo JJ, Meng MD. The recycled self: consumers’ disposal decisions of identity-linked products. J Consum Res. 2016;43(2):246–64.

[74] Van der Werff E, Steg L, Keizer K. The value of environmental self-identity: The relationship between biospheric values, environmental self-identity and environmental preferences, intentions and behaviour. J Environ Psychol. 2013;34:55–63.

[75] Tung T, Koenig H, Chen H. Effects of green self-identity and cognitive and affective involvement on patronage intention in eco-friendly apparel consumption: A gender comparison. Sustainability. 2017;9(11):1977.

[76] Cao J, Qiu H, Morrison AM. Self-Identity Matters: An Extended Theory of Planned Behavior to Decode Tourists’ Waste Sorting Intentions. Int J Environ Res Public Health. 2023;20(6):5099. doi: 10.3390/ijerph20065099 36982009 PMC10049705

[77] Thøgersen J, Møller B. Breaking car use habits: The effectiveness of a free one-month travelcard. Transp. 2008;35(3):329–45.

[78] Ganesan AS. Consumption, spending and investment behaviour of Malaysia Generation Y. (Master of Business Administration), Universiti Tunku Abdul Rahman, 2017. Retrieved from http://eprints.utar.edu.my/683/1/MBA-2012-08UKM1961-1.pdf

[79] Steg L, De Groot JI, Dreijerink L, Abrahamse W, Siero F. General antecedents of personal norms, policy acceptability, and intentions: The role of values, worldviews, and environmental concern. Soc Nat Resour. 2011;24(4):349–67.

[80] Lee K. Gender differences in Hong Kong adolescent consumers’ green purchasing behavior. Journal of Consumer Marketing. 2009;26(2):87–96. doi: 10.1108/07363760910940456

[81] Venhoeven LA, Bolderdijk JW, Steg L. Why Acting Environmentally-Friendly Feels Good: Exploring the Role of Self-Image. Front Psychol. 2016;7:1846. doi: 10.3389/fpsyg.2016.01846 27933017 PMC5121119

[82] Agarwal S. Impact of green computing in it industry to make eco-friendly environment. J Glob Res Comput Sci. 2014;5(4):5–10.

[83] Chetty M, Brush AB, Meyers BR, Johns P. It’s not easy being green: understanding home computer power management. Paper presented at the SIGCHI conference on human factors in computing systems, Boston, MA. 2009.

[84] Whitley C, Takahashi B, Zwickle A, Besley J, Lertpratchya A. Sustainability behaviors among college students: an application of the VBN theory. Environ Educ Res. 2018;24(2):245–62. doi: 10.1080/13504622.2016.1250151

[85] Kline R. Principles and practice of structural equation modeling. 3 ed. New York: The Guilford Press; 2010.

[86] Collier J. Applied structural equation modeling using AMOS: basic to advanced techniques. Routledge; 2020.

[87] Sathyanarayana S, Mohanasundaram T. Fit indices in structural equation modeling and confirmatory factor analysis: reporting guidelines. Asian J Econ Bus Account. 2024;24(7):561–77.

[88] Sahin E. Predictors of Turkish elementary teacher candidates’ energy conservation behaviors: An approach on value-belief-norm theory. Int J Environ Sci Educ. 2013;8(2):269–83.

[89] Raykov T. Bias-corrected estimation of non-centrality parameters of covariance structure models. Struct Equ Model. 2005;12(1):120–9.

[90] Winingsih PM, Rahmayanti H, Budiaman B, Miarsyah M. Norm activation model variable relationship: awareness of consequences, ascription of responsibility and personal norm. J Penelit Pendidik IPA. 2022;8(3):1273–9.

[91] Hu L, Bentler P. Cutoff criteria for fit indexes in covariance structure analysis: conventional criteria versus new alternatives. Struct Equ Modeling. 1999;6(1):1–55.

[92] Sharma R, Gupta A. Pro-environmental behaviour among tourists visiting national parks: application of value-belief-norm theory in an emerging economy context. Asia Pac J Tour Res. 2020;25(8):829–40.

[93] EPEAT. About EPEAT. 2021. Retrieved from https://www.epeat.net/about-epeat

[94] Dursun I, Kabadayi ET, Tuger AT. Application of value-belief-norm theory to responsible post consumption behaviors: recycling and reuse. In: Proceeding book. International Congress of the New Approaches and Technologies for Sustainable Development; 2017, 21–4.

[95] Jaini A, Quoquab F, Mohammad J, Hussin N. Antecedents of green purchase behavior of cosmetics products: An empirical investigation among Malaysian consumers. Int J Ethics Syst. 2020;36(2):185–203.

[96] Welfens M, Nordmann J, Seibt A. Drivers and barriers to return and recycling of mobile phones. Case studies of communication and collection campaigns. J Clean Prod. 2016;132(1):108–21. doi: 10.1016/j.jclepro.2015.11.082

[97] Zhang Z, Chen H, Dai J, Tan S, Zhang H. From biospheric values to tourists’ ecological compensation behavioural willingness: a comprehensive model test based on the value-identity-personal norm and normative activation. J Sustain Tour. 2024;32(12):2540–59.

[98] Zhao C, Zhang M, Wang W. Exploring the influence of severe haze pollution on residents’ intention to purchase energy-saving appliances. J Clean Prod. 2019;212(1):1536–43. doi: 10.1016/j.jclepro.2018.12.134

