# Supplementary material for: Comparing the antecedents of green computer behavior at acquisition, use, and disposal consumption stages from the moral norm and consumer attributes perspectives
Source: PLoS One. 2025 Jun 3;20(6):e0323622. doi: 10.1371/journal.pone.0323622 (PMC12132929; doi:10.1371/journal.pone.0323622)
Supplement: S1 Appendix — (DOCX) [file pone.0323622.s001.docx]

**S1 Appendix A. Construct Measures for the Computer Acquisition Phase**

|  | **Responsible Computer Acquisition (dependent factor)** |  |
| --- | --- | --- |
| RCA1 | I have bought energy efficient (eco-friendly) computers. | Agarwal (2014); Murugesan (2008) |
| RCA2 | I have referred to Electronic Product Environmental Assessment Tool (EPEAT) before making purchase decisions. |  |
| RCA3 | I have bought a computer with consideration of its negative impacts on the environment at the end of its life cycle. |  |
|  | **Habit (independent factor)** |  |
| HA1 | Responsible-computer purchase has become a habit for me. | Venkatesh et al. (2012) |
| HA2 | Practising green computer purchase has become natural to me. |  |
| HA3 | I must practise green computer purchase. |  |
|  | **Environmental knowledge (independent factor)** |  |
| EKA1 | I am knowledgeable about how to acquire green computers | Lee (2010) |
| EKA2 | I can explain what is meant by environmentally friendly computers |  |
| EKA3 | I can list at least three ways of acquiring environmentally friendly computers |  |
|  | **Self-identity (independent factor)** |  |
| SSA1 | I feel better than others if I practise green computer purchase | Lee (2009) |
| SSA2 | It is important to me to be known as someone who practises green computer purchase |  |
| SSA3 | Green computer purchase will enhance my self-image. |  |
| SSA4 | My involvement in green computer purchase is a status symbol. |  |
|  | **Biospheric value (independent factor from the VBN framework)** |  |
| Bio1 | Preventing pollution | Steg et al. (2005); Stern et al. (1999) |
| Bio2 | Respecting the earth |  |
| Bio3 | Unity with nature |  |
| Bio4 | Protecting the environment |  |
|  | **Environmental concern (independent factor from the VBN framework)** |  |
| EC1 | The so-called “ecological crisis” facing humankind has been greatly exaggerated. | Steg et al. (2005); Stern et al. (1999) |
| EC2 | The earth is like a spaceship with limited room and resources. |  |
| EC3 | If things continue their present course, we will soon experience a major ecological catastrophe. |  |
| EC4 | The balance of nature is strong enough to cope with the impacts of modern industrial nation. |  |
| EC5 | Mankind is severely abusing the environment. |  |
|  | **Awareness of Consequences (independent factor from the VBN framework)** |  |
| ACA1 | Climate change (greenhouse effects) will be a serious problem for other species of plants and animals. | Steg et al. (2005); Stern et al. (1999) |
| ACA2 | The problems of toxic substances in air and water and resulted from not practising green computer purchase behavior will be a serious problem to other species of plants and animals. |  |
| ACA3 | The depletion of resources due to not practising green computer purchase behavior will be a serious problem for me and my family. |  |
|  | **Ascription of Responsibility (independent factor from the VBN framework)** |  |
| ARA1 | I feel jointly responsible for greenhouse effects due to not practising green computer purchase behavior. | Steg et al. (2005); Stern et al. (1999) |
| ARA2 | I feel responsible for the presently occurring environmental problems due to not practising green computer purchase behavior. |  |
| ARA3 | I feel responsible for the depletion of energy resources due to not practising green computer purchase behavior. |  |
|  | **Personal Norms (independent factor from the VBN framework)** |  |
| PNA1 | I feel strong personal obligation to practise green computer purchase behavior. | \| Steg et al. (2005)tern et al. (1999) \| \| --- \| \| \| |
| PNA2 | I am willing to put extra effort into practising green computer purchase behavior. |  |
| PNA3 | I would feel guilty if I didn’t practise green computer purchase behavior. |  |
