# Supplementary material for: Comparing the antecedents of green computer behavior at acquisition, use, and disposal consumption stages from the moral norm and consumer attributes perspectives
Source: PLoS One. 2025 Jun 3;20(6):e0323622. doi: 10.1371/journal.pone.0323622 (PMC12132929; doi:10.1371/journal.pone.0323622)
Supplement: S2 Appendix — (DOCX) [file pone.0323622.s002.docx]

**S1 Appendix B. Construct Measures for the Computer Use Phase**

|  | **Responsible Computer Use (dependent factor)** |  |
| --- | --- | --- |
| RCU1 | I have turned off my computer when not in use. | Chetty et al. (2009); Murugesan (2008) |
| RCU3 | I have used lower power consumption mode, such as shutdown, hibernation, sleep or standby mode, when the computers are not in use. |  |
| RCU4 | I have used computers responsibly. |  |
|  | **Habit (independent factor)** |  |
| HU1 | Responsible use of computers has become a habit for me. | Venkatesh et al. (2012) |
| HU2 | Practising green use has become natural to me. |  |
| HU3 | I must practise green use. |  |
|  | **Environmental knowledge (independent factor)** |  |
| EKU1 | I am knowledgeable about how to use computers to protect the environment. | Lee (2010) |
| EKU2 | I can list at least three ways of using computers responsibly to protect the environment in our daily lives. |  |
| EKU3 | I often read to obtain more information about how to use computers to save the environment. |  |
|  | **Self-identity (independent factor)** |  |
| SSU1 | I feel better than others if I practise green use of computers. | Lee (2009) |
| SSU2 | It is important to me to be known as someone who practises green use of computers. |  |
| SSU3 | Green use will enhance my self-image. |  |
| SSU4 | My involvement in green use is a status symbol. |  |
|  | **Biospheric value (independent factor from the VBN framework)** |  |
| Bio1 | Preventing pollution |  |
| Bio2 | Respecting the earth | Steg et al. (2005); Stern et al. (1999) |
| Bio3 | Unity with nature |  |
| Bio4 | Protecting the environment |  |
|  | **Environmental concern (independent factor from the VBN framework)** |  |
| EC1 | The so-called “ecological crisis” facing humankind has been greatly exaggerated. |  |
| EC2 | The earth is like a spaceship with limited room and resources. | Steg et al. (2005); Stern et al. (1999) |
| EC3 | If things continue their present course, we will soon experience a major ecological catastrophe. |  |
| EC4 | The balance of nature is strong enough to cope with the impacts of modern industrial nation. |  |
| EC5 | Mankind is severely abusing the environment. |  |
|  | **Awareness of Consequences (independent factor from the VBN framework)** |  |
| ACU1 | Climate change (greenhouse effects) resulted from not practicing green use will be a serious problem for other species of plants and animals. | Steg et al. (2005); Stern et al. (1999) |
| ACU2 | The problems of toxic substances in air, water and resulted from not practising green use will be a serious problem to other species of plants and animals. |  |
| ACU3 | The depletion of resources due to not practising green use will be a serious problem for other species of plants and animals. |  |
|  | **Ascription of Responsibility (independent factor from the VBN framework)** |  |
| ARU1 | I feel jointly responsible for greenhouse effects due to not practising green use. | Steg et al. (2005); Stern et al. (1999) |
| ARU2 | I feel responsible for the presently occurring environmental problems due to not practising green use. |  |
| ARU3 | I feel responsible for the depletion of energy resources due to not practising green use. |  |
|  | **Personal Norms (independent factor from the VBN framework)** |  |
| PNU1 | I feel strong personal obligation to practise green use. | Steg et al. (2005); Stern et al. (1999) |
| PNU2 | I am willing to put extra effort into practising green use. |  |
| PNU3 | I would feel guilty if I didn't practise green use. |  |
