# Supplementary material for: Comparing the antecedents of green computer behavior at acquisition, use, and disposal consumption stages from the moral norm and consumer attributes perspectives
Source: PLoS One. 2025 Jun 3;20(6):e0323622. doi: 10.1371/journal.pone.0323622 (PMC12132929; doi:10.1371/journal.pone.0323622)
Supplement: S3 Appendix — (DOCX) [file pone.0323622.s003.docx]

**S1 Appendix C. Construct Measures for the Computer Disposal Phase**

|  | **Responsible Computer Disposal (dependent factor)** |  |
| --- | --- | --- |
| RCD1 | I have reused my old computer or part of it since it still met my requirement. | Murugesan (2008); Williams and Sasaki (2003) |
| RCD3 | I have upgraded or refurbished old computer to meet my new requirements instead of buying new computer for replacement. |  |
| RCD4 | I have recycled components of used computer. |  |
| RCD5 | I have disposed of computer responsibly. |  |
| RCD6 | I resold my computer via the internet or other means. |  |
|  | **Habit (independent factor)** |  |
| HD1 | Responsible disposal of computers has become a habit for me. | Venkatesh et al. (2012) |
| HD2 | Practising green disposal has become natural to me. |  |
| HD3 | I must practise green disposal. |  |
|  | **Environmental knowledge (independent factor)** |  |
| EKD1 | I am knowledgeable about how to dispose of computers responsibly to protect the environment. | Lee (2010) |
| EKD2 | I can explain what is meant by recycling. |  |
| EKD3 | I can list at least three ways of disposing of old computers responsibly. |  |
|  | **Self-identity (independent factor)** |  |
| SSD1 | I feel better than others if I practice green disposal of computers. | Lee (2009) |
| SSD2 | It is important to me to be known as someone who practises green disposal of computers. |  |
| SSD3 | Green disposal will enhance my self-image. |  |
|  | **Biospheric value (independent factor from the VBN framework)** |  |
| Bio1 | Preventing pollution |  |
| Bio2 | Respecting the earth | Steg et al. (2005); Stern et al. (1999) |
| Bio3 | Unity with nature |  |
| Bio4 | Protecting the environment |  |
|  | **Environmental concern (independent factor from the VBN framework)** |  |
| EC1 | The so-called “ecological crisis” facing humankind has been greatly exaggerated. |  |
| EC2 | The earth is like a spaceship with limited room and resources. | Steg et al. (2005); Stern et al. (1999) |
| EC3 | If things continue their present course, we will soon experience a major ecological catastrophe. |  |
| EC4 | The balance of nature is strong enough to cope with the impacts of modern industrial nation. |  |
| EC5 | Mankind is severely abusing the environment. |  |
|  | **Awareness of Consequences (independent factor from the VBN framework)** |  |
| ACD1 | Climate change (greenhouse effects) resulted from not practising green disposal will be a serious problem for me and my family. | Steg et al. (2005); Stern et al. (1999) |
| ACD2 | The problems of toxic substances in air, water and resulted from not practising green disposal will be a serious problem for me and my family. |  |
| ACD3 | The depletion of resources due to not practising green disposal will be a serious problem for me and my family. |  |
|  | **Ascription of Responsibility (independent factor from the VBN framework)** |  |
| ARD1 | I feel jointly responsible for greenhouse effects due to not practising green disposal. | Steg et al. (2005); Stern et al. (1999) |
| ARD2 | I feel responsible for the presently occurring environmental problems due to not practising green disposal. |  |
| ARD3 | I feel responsible for the depletion of energy resources due to not practising green disposal. |  |
|  | **Personal Norms (independent factor from the VBN framework)** |  |
| PND1 | I feel strong personal obligation to practise green disposal. | Steg et al. (2005); Stern et al. (1999) |
| PND2 | I am willing to put extra effort into practising green disposal. |  |
| PND3 | I would feel guilty if I didn't practise green disposal. |  |
