# Supplementary material for: Comparing the antecedents of green computer behavior at acquisition, use, and disposal consumption stages from the moral norm and consumer attributes perspectives
Source: PLoS One. 2025 Jun 3;20(6):e0323622. doi: 10.1371/journal.pone.0323622 (PMC12132929; doi:10.1371/journal.pone.0323622)
Supplement: S4 Appendix — (DOCX) [file pone.0323622.s004.docx]

**S1 Appendix D. Comparing Differences between Groups in the Computer Acquisition Phase**

**a) Gender**

Table 1a: t-Test (assuming equal variance) results for gender differences

| **Gender** | **Mean** | **SD** | **t-value** | **df** | **p-value** |
| --- | --- | --- | --- | --- | --- |
| Male | 3.059 | 0.892861 |  |  |  |
| Female | 3.133 | 0.846702 | 1.963 | 919 | 0.201 |

The results show that there is no statistically significant between males and females regarding computer acquisition.

**b) Age**

Table 1b: One-way ANOVA test to compare differences among age groups.

| **Source of Variation** | **SS** | **df** | **MS** | **F** | **P-value** | **F crit** |
| --- | --- | --- | --- | --- | --- | --- |
| Between Groups | 4.948 | 2 | 2.474 | 3.269 | 0.039 | 3.006 |
| Within Groups | 694.800 | 918 | 0.757 |  |  |  |
| Total | 699.747 | 920 |  |  |  |  |

The results show that there are statistically significant differences at 0.05 among the 3 age groups (17 – 32,

33 – 47, 48 and over) regarding computer acquisition.

**c) Income**

Table 1c: One-way ANOVA test to compare differences among income groups.

| **Source of Variation** | **SS** | **df** | **MS** | **F** | **P-value** | **F crit** |
| --- | --- | --- | --- | --- | --- | --- |
| Between Groups | 3.284 | 3 | 1.095 | 1.441 | 0.229 | 2.615 |
| Within Groups | 696.464 | 917 | 0.760 |  |  |  |
| Total | 699.747 | 920 |  |  |  |  |

The results show that there are no statistically significant differences among the income groups (0 – 2,000, 2,001 – 4,000, 4,001 – 6,000, Over 6,000) regarding computer acquisition.

**d) Education**

Table 1d: One-way ANOVA test for comparing differences among education level groups.

| **Source of Variation** | **SS** | **df** | **MS** | **F** | **P-value** | **F crit** |
| --- | --- | --- | --- | --- | --- | --- |
| Between Groups | 8.646 | 5 | 1.729 | 2.289 | 0.044 | 2.224 |
| Within Groups | 691.102 | 915 | 0.755 |  |  |  |
| Total | 699.747 | 920 |  |  |  |  |

The results show that there are statistically significant differences at 0.05 among the education groups (Secondary and below, Pre-university, Diploma, Degree, Postgraduate, Others) regarding computer acquisition.
