# Supplementary material for: Comparing the antecedents of green computer behavior at acquisition, use, and disposal consumption stages from the moral norm and consumer attributes perspectives
Source: PLoS One. 2025 Jun 3;20(6):e0323622. doi: 10.1371/journal.pone.0323622 (PMC12132929; doi:10.1371/journal.pone.0323622)
Supplement: S5 Appendix — (DOCX) [file pone.0323622.s005.docx]

**S1 Appendix E. Comparing differences between Groups in the Computer Use Phase**

**a) Gender**

Table 3a: t-Test (assuming equal variance) results for gender differences.

| **Gender** | **Mean** | **SD** | **t-value** | **df** | **p-value** |
| --- | --- | --- | --- | --- | --- |
| Male | 3.831 | 0.773 |  |  |  |
| Female | 3.978 | 0.733 | 1.963 | 919 | 0.003 |

The results show that there is statistically significant at 0.05 between males and females regarding green computer use.

**b) Age**

Table 3b: One-way ANOVA test to compare differences among age groups.

| **Source of Variation** | **SS** | **df** | **MS** | **F** | **P-value** | **F crit** |
| --- | --- | --- | --- | --- | --- | --- |
| Between Groups | 1.680 | 2 | 0.840 | 1.464 | 0.232 | 3.006 |
| Within Groups | 526.917 | 918 | 0.574 |  |  |  |
| Total | 528.597 | 920 |  |  |  |  |

The results show that there is no statistically significant differences among the age groups regarding green computer use.

**c) Income**

Table 3c: One-way ANOVA test to compare differences among income groups.

| **Source of Variation** | **SS** | **df** | **MS** | **F** | **P-value** | **F crit** |
| --- | --- | --- | --- | --- | --- | --- |
| Between Groups | 7.009 | 3 | 2.336 | 4.107 | 0.007 | 2.615 |
| Within Groups | 521.588 | 917 | 0.569 |  |  |  |
| Total | 528.597 | 920 |  |  |  |  |

The results show that there is statistically significant at 0.05 among the income levels regarding green computer use.

**d) Education**

Table 3d: One-way ANOVA test for comparing differences among education level groups.

| **Source of Variation** | **SS** | **df** | **MS** | **F** | **P-value** | **F crit** |
| --- | --- | --- | --- | --- | --- | --- |
| Between Groups | 4.55 | 5 | 0.909 | 1.587 | 0.161 | 2.224 |
| Within Groups | 524.05 | 915 | 0.573 |  |  |  |
| Total | 528.60 | 920 |  |  |  |  |

The results show that there is no statistically significance among the education levels regarding green computer use.
