# Supplementary material for: Comparing the antecedents of green computer behavior at acquisition, use, and disposal consumption stages from the moral norm and consumer attributes perspectives
Source: PLoS One. 2025 Jun 3;20(6):e0323622. doi: 10.1371/journal.pone.0323622 (PMC12132929; doi:10.1371/journal.pone.0323622)
Supplement: S6 Appendix — (DOCX) [file pone.0323622.s006.docx]

**S1 Appendix F. Comparing differences between Groups in the Computer Disposal Phase**

**a) Gender**

Table 2a: t-Test (assuming equal variance) results for gender differences

| **Gender** | **Mean** | **SD** | **t-value** | **df** | **p-value** |
| --- | --- | --- | --- | --- | --- |
| Male | 3.233 | 0.895 |  |  |  |
| Female | 3.098 | 0.848 | 1.963 | 919 | 0.020 |

The results show that there is statistically significant at 0.05 between males and females regarding green computer disposal.

**b) Age**

Table 2b: One-way ANOVA test to compare differences among age groups.

| **Source of Variation** | **SS** | **df** | **MS** | **F** | **P-value** | **F crit** |
| --- | --- | --- | --- | --- | --- | --- |
| Between Groups | 20.813 | 2 | 10.407 | 13.957 | 0.000 | 3.006 |
| Within Groups | 684.479 | 918 | 0.746 |  |  |  |
| Total | 705.292 | 920 |  |  |  |  |

The results show that there is statistically significant at 0.05 between the age groups regarding green computer disposal.

**c) Income**

Table 2c: One-way ANOVA test to compare differences among income groups.

| **Source of Variation** | **SS** | **df** | **MS** | **F** | **P-value** | **F crit** |
| --- | --- | --- | --- | --- | --- | --- |
| Between Groups | 10.568 | 3 | 3.523 | 4.650 | 0.003 | 2.615 |
| Within Groups | 694.724 | 917 | 0.758 |  |  |  |
| Total | 705.292 | 920 |  |  |  |  |

The results show that there is statistically significant at 0.05 among the income levels regarding green computer disposal.

**d) Education**

Table 2d: One-way ANOVA test for comparing differences among education level groups.

| **Source of Variation** | **SS** | **df** | **MS** | **F** | **P-value** | **F crit** |
| --- | --- | --- | --- | --- | --- | --- |
| Between Groups | 5.485 | 5 | 1.097 | 1.434 | 0.209 | 2.224 |
| Within Groups | 699.807 | 915 | 0.765 |  |  |  |
| Total | 705.292 | 920 |  |  |  |  |

The results show that there is no statistically significance among the education levels regarding green computer disposal.
