# Supplementary material for: Comparing the antecedents of green computer behavior at acquisition, use, and disposal consumption stages from the moral norm and consumer attributes perspectives
Source: PLoS One. 2025 Jun 3;20(6):e0323622. doi: 10.1371/journal.pone.0323622 (PMC12132929; doi:10.1371/journal.pone.0323622)
Supplement: S2 File — (PDF) [file pone.0323622.s008.pdf]

MONASH University

Monash University Human Research Ethics Committee (MUHREC)
Research Office

Human Ethics Certificate of Approval

**Date:**	**01 November 2012**
**Project Number:**	**CF12/3196 – 2012001588**
**Project Title:**	**Building and Validating a Model for Responsible Consumption Behaviour**
**Chief Investigator:**	**Assoc Prof Paul P. H. Yeow**
**Approved:**	**From: 01 November 2012 To: 01 November 2017**

Terms of approval

1. The Chief investigator is responsible for ensuring that permission letters are obtained, if relevant, and a copy forwarded to MUHREC before any data collection can occur at the specified organisation. **Failure to provide permission letters to MUHREC before data collection commences is in breach of the National Statement on Ethical Conduct in Human Research and the Australian Code for the Responsible Conduct of Research.**
2. Approval is only valid whilst you hold a position at Monash University.
3. It is the responsibility of the Chief Investigator to ensure that all investigators are aware of the terms of approval and to ensure the project is conducted as approved by MUHREC.
4. You should notify MUHREC immediately of any serious or unexpected adverse effects on participants or unforeseen events affecting the ethical acceptability of the project.
5. The Explanatory Statement must be on Monash University letterhead and the Monash University complaints clause must contain your project number.
6. **Amendments to the approved project (including changes in personnel):** Requires the submission of a Request for Amendment form to MUHREC and must not begin without written approval from MUHREC. Substantial variations may require a new application.
7. **Future correspondence:** Please quote the project number and project title above in any further correspondence.
8. **Annual reports:** Continued approval of this project is dependent on the submission of an Annual Report. This is determined by the date of your letter of approval.
9. **Final report:** A Final Report should be provided at the conclusion of the project. MUHREC should be notified if the project is discontinued before the expected date of completion.
10. **Monitoring:** Projects may be subject to an audit or any other form of monitoring by MUHREC at any time.
11. **Retention and storage of data:** The Chief Investigator is responsible for the storage and retention of original data pertaining to a project for a minimum period of five years.

Ben Canny

Professor Ben Canny
Chair, MUHREC

cc: Dr Uchenna Cyril Eze, Ms Loo Wee Hong

Postal – Monash University, Vic 3800, Australia
Building 3E, Room 111, Clayton Campus, Wellington Road, Clayton
Telephone +61 3 9905 5490 Facsimile +61 3 9905 3831
Email muhrec@monash.edu www.monash.edu/research/ethics/human/index/html
ABN 12 377 614 012 CRICOS Provider #00008C
